# Lactopeptine

**Published:** 1880-09

**Authors:** 


LACTOPEPTINE.—We have no hesitation in calling attention to this excellent adjuvant in the treatment of the gastro-intestinal troubles, that are prostrating so large a number of the infant population, and hastening many of them to their early graves, during the present hot weather, in our cities and towns. Experience with this article during the present "heated term," in the treatment of the so-called "summer complaints," of infants and children, enables us to speak of it in high terms of commendation, and as the most valuable auxiliary to the remedies we have prescribed for this class of sufferers, at any time.

A sample of Lactopeptine was sent us from New York, by the company engaged in its manufacture, early in the season, but it was some time before we began its use in practice; and it is but simple justice to say, it has more than realized *all* we hoped for, or expected from its use, thus far.

